# The influence of weight-bearing status on post-operative mobility and outcomes in geriatric hip fracture

**DOI:** 10.1007/s00068-022-01939-6

**Published:** 2022-03-15

**Authors:** Seth Michael Tarrant, John Attia, Zsolt Janos Balogh

**Affiliations:** 1 Department of Traumatology, John Hunter Hospital, Lookout Rd, New Lambton Heights, NSW 2305 Australia; 2 School of Medicine and Public Health, University of Newcastle, Callaghan, NSW 2308 Australia; 3 Hunter Medical Research Institute, New Lambton Heights, NSW 2305 Australia; 4 Trauma Service, Division of Surgery, John Hunter Hospital, Hunter Region Mail Centre, Locked Bag No. 1, New Lambton Heights, NSW 2310 Australia

**Keywords:** Hip fracture, Mobility, Weight-bear, Mortality, Outcomes

## Abstract

**Purpose:**

We hypothesized that unrestricted or full weight-bearing (FWB) in hip fracture would increase the opportunity to mobilize on post-operative day 1 (POD1mob) and be associated with better outcomes compared with restricted weight-bearing (RWB).

**Methods:**

Over 4 years, 1514 geriatric hip fracture patients aged 65 and above were prospectively recruited. Outcomes were compared between FWB and RWB patients. The primary outcome was 30-day mortality. Secondary outcomes were immobility-related adverse events, length of stay (LOS), and reoperation for failure. Causal effect modelling and multivariate regression with mediation analyses were performed to examine the relation between weight-bearing status (WBS), POD1mob, and known mortality predictors.

**Results:**

FWB was allowed in 1421 (96%) of 1479 surgically treated patients and RWB enforced in 58 (4%) patients. Mortality within 30 days occurred in 141 (9.9%) of FWB and 3 (5.2%) of RWB patients. In adjusted analysis, RWB did not influence 30-day mortality (OR 0.42, 95% CI 0.15–01.13, *p* = 0.293), with the WBS accounting for 91% of the total effect on mortality and 9% contributed from how WBS influenced the POD1mob. RWB was significantly related to increased DVT (OR 7.81, 95% CI: 1.81–33.71 *p* = 0.002) but no other secondary outcomes. Patients that did not have the opportunity to mobilize had increased 30-day mortality (OR 2.31, 95% CI 1.53–3.48 *p* < 0.001).

**Conclusion:**

Restricted weight-bearing was not associated with increased 30-day mortality. Only a small proportion of this effect was mediated by POD1mob. Whilst post-surgical WBS may be difficult to influence for cultural reasons, POD1mob is an easily modifiable target that is likely to have a greater effect on 30-day mortality.

**Level of evidence:**

Level III, observational study.

## Introduction

Osteoporosis will become an epidemic in the developed world with geriatric hip fractures estimated to increase, particularly in the Asia–Pacific region [1]. Whilst guidelines encourage early mobilization ubiquitously, the Australian & New Zealand Hip Fracture Registry (ANZFHR) is one of only a few registries to capture unrestricted or full weight-bearing (FWB) as a data field [2]. Improving mobility in hip fracture is difficult [3], but consensus is that FWB can facilitate this. Despite strong recommendations for FWB, there is a paucity of evidence on how weight-bearing status (WBS) affects outcomes. The ANZHFR goal of FWB is not fully achieved, with approximately 5% of patients restricted or ‘not known’ [4]. This is unlike the recent data from the United States where 25% of hip fractures are restricted weight-bearing (RWB) [5]. Whilst there are some reports that RWB has led to longer length of stay [6], post-operative adverse events, and increased 30-day mortality [5], external validity and generalizability of these results require investigation.

This aim of this single centre study is to compare demographics, injury patterns, treatment factors, and outcomes between patients with RWB and FWB status. Due to the assumed intrinsic link between WBS and the ability to mobilize immediately post-operatively, we hypothesized that FWB and its effect on post-operative mobilization would be associated with better outcomes compared with restricted weight-bearing.

## Methods

### Patients and setting

Patients aged 65 and above with a low-energy hip fracture were recruited from the Australia New Zealand Hip Fracture Registry (ANZHFR) at the John Hunter Hospital, a Level 1 Tertiary referral major trauma centre located in the Australian state of New South Wales, as approved by local ethics governance (HREC/14/CIPHS/51). Data are collected by experienced specialist clinical nurses according to a defined minimum data set [7]. Exclusion criteria were high-energy injury, pathological fracture (not including osteoporosis), and periprosthetic or peri-implant fracture.

Patients admitted between February 2015 and December 2018 were identified. Demographics (age, sex, residence, pre-injury mobility, cognitive impairment), peri-operative data (American Society of Anesthesiology [ASA] score, operative intervention), the opportunity to mobilize on post-operative day 1 (POD1mob) and discharge destination were taken from the registry.

### Weight-bearing status

Weight-bearing status (WBS) is recorded in the ANZHFR as either: (1) “unrestricted weight bearing”, which “refers to a patient who is able to mobilize with full use of the affected limb to weight bear as pain allows” (the acronym FWB represents this); (2) “restricted/non weight-bearing” (RWB) which refers to “a patient where there is a specific instruction that prevents the patient being allowed to fully utilize the leg irrespective of degree of pain. Restricted weight-bearing includes terms such as partial weight bear, touch‐weight bear and non-weight-bear.”; and (3) “not known” [7]. Registry-reported WBS was retrospectively cross checked with the operative orders and throughout the course of admission. Due to the well-established inability of geriatric patients to limit weight-bearing [8], all WBS fields that were not FWB were considered RWB.

### Fracture type

Fracture types recorded by the ANZHFR consist of four options: non-displaced femoral neck fractures, displaced femoral neck fractures, intertrochanteric fractures (including basicervical), and subtrochanteric fracture. All radiographs of the patients with RWB were retrospectively classified using the AO system (Arbeitsgemeinschaft für Osteosynthesefragen) [9].

### Outcomes

The primary outcome was 30-day mortality recorded in the ANZHFR. Mortality was cross-referenced with the New South Wales Registry of Births, Deaths, and Marriages. Secondary outcomes were length of acute orthopaedic stay (LOS), immobility-related adverse events, and revision surgery at 30 days and 120 days. Infection-related causes for revision were excluded. A panel of immobility-related adverse events were examined in the cohort; these included pulmonary embolus (PE), deep vein thrombosis (DVT), lower respiratory tract infection (LRTI), delirium, urinary tract infection (UTI), and a post-operative fall on the ward as an inpatient. PE was confirmed usually on a computed tomography pulmonary angiogram (CTPA) but occasionally on a V/Q (ventilation/perfusion) nuclear scan when CTPA was contraindicated due to poor renal function. Deep Vein Thrombosis (DVT) was diagnosed after clinical suspicion (not routine testing) by a qualified vascular-trained ultrasonographer. LRTI, or pneumonia, was diagnosed with a clinical picture of dyspnoea and fever with pathological findings on chest radiographic films. Delirium was a clinical diagnosis made by the treating Orthogeriatric team by DSM-5 criteria. UTI was defined by positive post-operative culture. Falls on the ward were documented by the nursing staff.

### Statistics

**Fig. 1:**
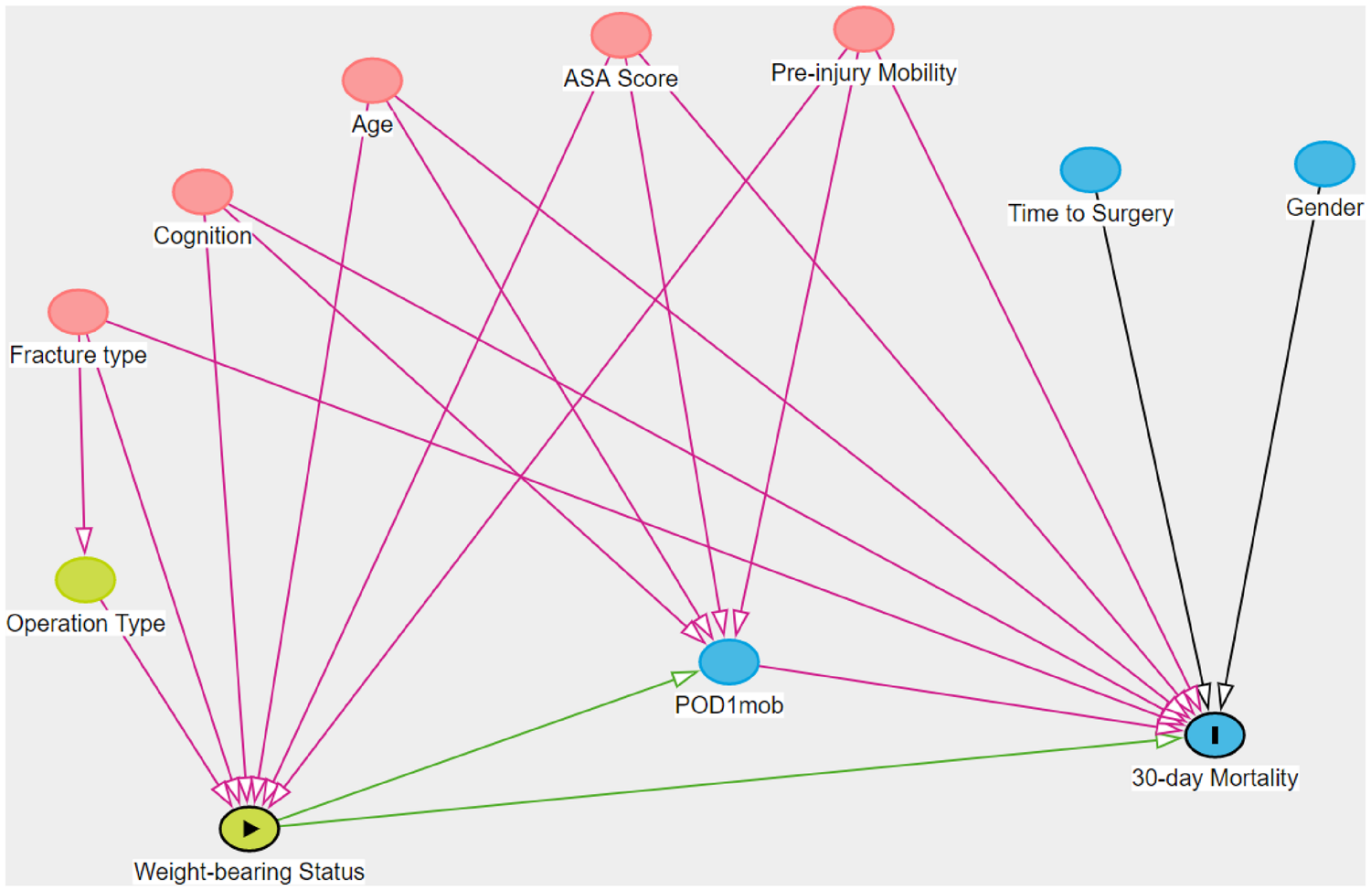
Causal effect model of WBS (exposure) and 30-day mortality (outcome)

Continuous data were assessed for distribution and reported as mean with standard deviation if parametric, or median with 1st and 3rd quartile ranges if non-parametric. Categorical data were expressed as counts and percentages. Normally distributed data were assessed with independent *t* test and non-parametric analyses were performed using Mann–Whitney *U* test. For categorical data, Chi^2^ was used. Univariate analyses for mortality were performed with logistic regression; however, this was not used to identify model variables so as to reduce the risk of data-driven predictor selection bias [10]. Causal modelling was performed to define confounders that influenced both the treatment (WBS) and outcomes (Fig. 1). The confounders were chosen a priori from our Institution’s previous publication on independent risk factors [11] for 30-day mortality. From the model, the minimal sufficient adjustment sets for estimating the direct effect of WBS on 30-day mortality included the independent variables of ASA score, age, cognitive impairment, POD1mob, fracture type, and pre-injury mobility. Logistic regression with mediation analysis was used to account for the theoretical relationship that restricted weight-bearing will affect the ability to POD1mob, which has been associated with increased 30-day mortality [12]. The mediation analysis decomposed the total effect of RWB on 30-day mortality into 2 components: the natural indirect effect size (i.e., the effect size of RWB due to mediation through POD1mob), and the natural direct effect size (i.e., the effect size of RWB not explained through the mediator). The effect on 30-day mortality mediated by POD1m was calculated along with other confounders and presented as odds ratios with bootstrapping (1000 iterations) of the 95% confidence intervals (CI). The proportion of mediation was calculated on the natural logarithm scale and given as a percentage: ln(ORindirect effect)/ln(ORtotal effect) × 100.

For immobility-related adverse events, the same model was used (Fig. 1). For the non-parametric continuous LOS variable with a binary mediator and binary treatment group, Poisson and logistic regression was used for mediation analysis. Variables were the same as Fig. 1 with time to surgery and pre-injury nursing home status included. For revision surgery, multivariate logistic regression with operation type and without mediation from POD1mob was used, as it was theorised not to affect the overall need for revision surgery.

A sub-analysis of POD1mob was conducted after causal modelling (Appendix 2). Binary outcomes of 30-day mortality and immobility-related adverse events were assessed with multivariate logistic regression. Generalised linear modelling using log transformation and gamma regression was used for LOS.

The alpha value was set at 0.05. Statistical analysis was conducted with Stata v13.0 (StataCorp, College Station, TX).

## Results

### Demographics

A total of 1,514 patients were identified during the study period. The average age of the population was 83.5 (± 9.7 SD) years, with 1046 (69%) female, 379 (25%) from a nursing home, and 599 (40%) with known cognitive impairment. Regarding pre-morbid mobility, 649 (43%) were independently mobile, 199 (13%) used a walking stick, 613 (41%) used a frame, and 49 (3.3%) were restricted to a wheelchair or bed-bound.

Median orthopaedic length of stay was 7 (*Q*_1–3_: 4–11) days. Discharge destination was to home in 157 (10.5%) of patients, public rehabilitation in 324 (22%), private rehabilitation in 443 (30%), nursing home in 396 (27%), hospital/ward transfer in 95 (6.4%), and inpatient death in 78 (5.2%). Mortality within 30 days occurred in 154 patients (11%).

### Fracture type and operative intervention

Fracture types were subdivided by ANZHFR criteria into 372 (25%) displaced intracapsular femoral neck fractures, 330 (22%) undisplaced or impacted intracapsular femoral neck fractures, 746 (49%) trochanteric fractures (including basicervical patterns), and 66 (4.4%) subtrochanteric fractures.

Operative intervention occurred in 1,479 (98%) of patients. Time to surgery was a median of 1.0 days (Q_1-3_: 0.8–1.5). General Anaesthesia was used in 814 (55%) of patients. ASA was grade I in 11 (0.7%) patients, grade II in 216 (15%), grade III in 856 (60%), grade IV in 365 (25%), and grade V in 4 (0.3%).

### Post-operative weight-bearing

#### FWB and RWB

Of 1479 surgically treated patients, 1421 (96%) were FWB and 58 (4%) patients were RWB. Patients who were RWB were younger (*p* = 0.022) but displayed no difference in gender (*p* = 0.072), admission from a nursing home (*p* = 0.967), cognitive impairment (*p* = 0.272), pre-morbid mobility (*p* = 0.498), or ASA status (0.498) (Table 1).

**Table 1:** Comparison of weight-bearing status

	FWB (1421)	RWB (58)	*p* Value
Age (years; mean, SD)	83.8 (± 7.9)	81.3 (± 9.4)	0.022
Gender (female; *n*, %)	991 (70%)	34 (59%)	0.072
Residence (nursing home; *n*, %)	363 (26%)	15 (26%)	0.967
Cognitive impairment (impaired; *n*, %)	559 (40%)	19 (33%)	0.272
ASA (score; mean, SD)			0.498
1	11 (0.8%)	0 (0%)	
2	205 (15%)	10 (18%)	
3	819 (59%)	37 (67%)	
4	356 (26%)	9 (16%)	
5	4 (0.3%)	0 (0%)	
Mobility (type; *n*, %)			0.743
Independent	616 (43%)	25 (44%)	
One aid	187 (13%)	10 (18%)	
Frame	575 (40%)	21 (37%)	
Wheel chair/bed bound	42 (3.0%)	1 (1.8%)	
Fracture (ANZHFR type; *n*, %)			< 0.001
Undisplaced/impacted intracapsular	354 (25%)	13 (22%)	
Displaced intracapsular	290 (20%)	21 (36%)	
Per/intertrochanteric (including basicervical)	719 (51%)	17 (29%)	
Subtrochanteric	58 (4%)	7 (12%)	
Operations (type; *n*, %)			< 0.001
Cannulated screws (*n* = 129)	100 (6.9%)	29 (50%)	
Cemented hemiarthroplasty (*n* = 427)	425 (29%)	2 (3.5%)	
Uncemented hemiarthroplasty (*n* = 28)	28 (1.9%)	0 (0%)	
Long femoral nail (*n* = 246)	229(16%)	17 (29%)	
Short femoral nail (*n* = 504)	500 (34%)	4 (4.9%)	
Other (*n* = 9)	8 (0.5%)	1 (1.7%)	
Sliding hip screw (*n* = 55)	50 (3.4%)	5 (8.6%)	
Cemented total hip replacement (*n* = 78)	78 (5.4%)	0 (0%)	
Uncemented total hip replacement (*n* = 3)	3 (0.0%)	0 (0%)	

*ANZHFR* Australia and New Zealand Hip Fracture Registry, *ASA* American Society of Anesthesiologists, *FWB* full weight-bearing, *RWB* restricted weight-bearing, *SD* standard deviation

#### Fracture patterns and operation type with restricted weight-bearing

Analysis of fracture patterns according to the ANZHFR classification demonstrated that there was a significant difference between fracture types (*p* < 0.001) with ‘displaced femoral neck’ and ‘subtrochanteric’ fractures over-represented in the RWB group (Table 1). There was a significant difference in operation types between groups (*p* < 0.001) with a higher proportion of RWB patients receiving cannulated screws, long femoral nails, and sliding hip screws (Table 1).

When defined by the AO fracture pattern classification, the AO31B1.1 valgus-impacted femoral neck fracture pattern was found in 22 (38%) RWB patients, with 20 cannulated screws and 2 DHS constructs used in these patients (Table 1). The fracture pattern with the second highest proportion of restrictions was the AO31A3.3 intertrochanteric fracture with a reverse-oblique lateral wall component and separation of medial calcar accounting for 11 (19%) of fractures. All patients in this group received long intramedullary nails (Table 2).

**Table 2:** Restricted weight-bearing by AO fracture type

Fracture type	*n* = 58
31A (*n* = 20)	
1.2	2 (3.4%)
1.3	1 (1.7%)
2.2	1 (1.7%)
2.3	3 (5.2%)
3.1	2 (3.4%)
3.3	11 (19%)
31B (*n* = 36)	
1.1	22 (38%)
1.2	3 (5.2%)
1.3	7 (12%)
2.1	3 (5.2%)
2.2	1 (1.7%)
32A (*n* = 2)	
1.3	2 (3.4%)

*AO* Arbeitsgemeinschaft für Osteosynthesefragen

### Outcomes

#### 30-Day mortality

**Fig. 2:**
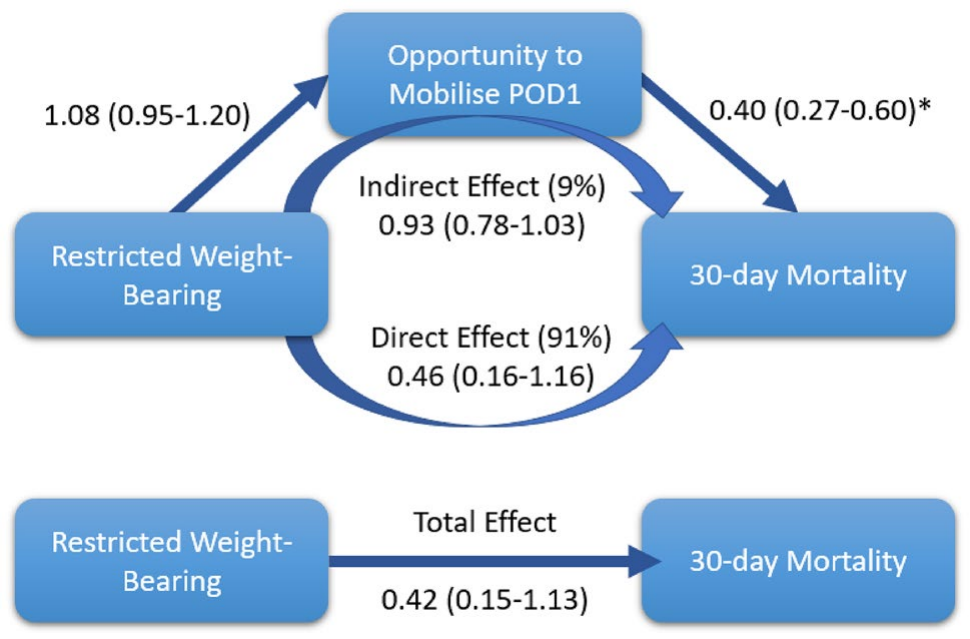
Fully adjusted mediation analyses presented as odds ratios with 95% confidence intervals. ‘*’ denotes *p* < 0.05

The 30-day mortality outcome occurred in 141 (9.9%) FWB and 3 (5.2%) RWB patients. From the causal model (Fig. 2), RWB had a non-significant effect on POD1mob; however, POD1mob had a significant effect on 30-day mortality (*p* < 0.001). Overall, RWB was not significantly associated with 30-day mortality (Fig. 2 and Table 3), with the weight-bearing status accounting for 91% of the total effect on mortality and 9% contributed from how weight-bearing influenced POD1mob (Fig. 2). In the adjusted model, demographics influencing mortality were cognitive impairment (adjusted odds ratio [aOR] 2.07, 95% CI 1.34–3.19, *p* = 0.001), a displaced femoral neck fracture (aOR 2.43, CI 95% 1.27–4.65, *p* = 0.007), or a trochanteric fracture (aOR 2.11, CI 95% 1.18–3.78, *p* = 0.012) compared with other fracture types. They did not affect POD1mob (displaced femoral neck fracture aOR 0.93, CI 95% 0.62–1.40, *p* = 0.750; trochanteric fractures—aOR 0.94, CI 95% 0.67–1.31, *p* = 0.719). Subtrochanteric fracture pattern had no effect on mortality (aOR 1.18, CI 95% 0.36–3.89, *p* = 0.782), but did have a lower chance of POD1mob (aOR 0.45, CI 95% 0.24–0.84, *p* = 0.782).

**Table 3:** Multivariate regression analysis of outcomes and restricted weight-bearing mediated by POD1mob

Variable	Outcome incidence		Total		
	FWB (1421)	RWB (58)	OR	95% CI	*p* value
Primary outcome					
30-Day mortality^a^	141 (9.9%)	3 (5.2%)	0.42	(0.15–1.13)	0.293
Secondary outcomes					
Adverse events (type; *n*, %)^a^					
Deep vein thrombosis	7 (0.5%)	2 (3.5%)	7.81	(1.81–33.71)	0.002
Lower respiratory tract infection	127 (8.9%)	4 (6.9%)	0.96	(0.30–3.11)	0.942
Delirium	329 (23%)	14 (24%)	0.83	(0.42–1.63)	0.583
Urinary tract infection	236 (17%)	13 (22%)	0.71	(0.32–1.59)	0.407
Falls on the ward	13 (0.9%)	2 (3.5%)	0.46	(0.14–1.47)	0.189
Pulmonary embolus	9 (0.6%)	0 (0%)	–		
Orthopaedic LOS (days; median, Q1–3)^a^	7 (4–11)	7 (4–11)	1.04	(0.94–1.15)	0.482
Reoperation (*n*, %)^b^					
Reoperation within 30 days	5 (0.3%)	1 (1.7%)	1.93	(0.16–23.04)	0.605
Reoperation within 120 days	16 (1.1%)	4 (6.9%)	2.03	(0.54–7.54)	0.293

*CI* confidence interval, *FWB* full weight-bearing, *OR* odds ratio, *POD1mob* opportunity to mobilized post-operative day 1, *RWB* restricted weight-bearing, *SE* standard error

^a^Adjusted for age, cognitive state, ASA score, fracture type and pre-injury mobility status

^b^Adjusted for age, cognitive state, ASA score, fracture type, operation type, and pre-injury mobility status

#### Secondary outcomes

Restricted weight-bearing was related to a significantly higher rate of DVT (*p* = 0.002); in decomposition into direct and indirect associations, a negative 1.5% of indirect effect was mediated by POD1mob, but was not significant (OR 0.97, 95% CI 0.82–1.15, *p* = 0.748) (Table 3). The other adverse events of LRTI, delirium, UTI, falls on the ward, and PE (no occurrences in RWB group) showed no difference between groups for total, indirect or direct effects (Table 3). Multivariate analysis demonstrated that RWB did not affect acute orthopaedic length of stay or the risk of reoperation at 30 days and 120 days (Table 3).

#### Sub-analysis of POD1mob

A sub-analysis of demographics and outcomes relating to POD1 mobility was performed as per causal modelling (Appendix 1) with minimal sufficient adjustment requiring age, ASA score, pre-injury mobility, and weight-bearing status. Demographics and outcomes are listed in Appendix 2. Patients not given the opportunity to mobilize day 1 were significantly older (*p* < 0.001), had more limited pre-injury mobility (*p* = 0.004), were more cognitively impaired (*p* < 0.001), and were more comorbid as indicated with a higher ASA score (*p* < 0.001). There was no difference in operation type nor time to surgery (appendix 2).

Patients not given the opportunity to mobilize had a higher 30-day mortality, but had no increased risk of immobility-related complications (Appendix 3).

## Discussion

This analysis has shown that RWB is not associated with an increased 30-day mortality. For secondary outcomes, RWB patients had a higher rate of DVT but no increased risk of other immobility-related adverse events. RWB did not influence LOS or reoperation in adjusted analysis. Patients not given the opportunity to mobilize on day 1 post-operatively were older, had worse pre-injury mobility, worse cognition, and more comorbidities, but had similar WBS. They showed no difference in LOS or immobility-related adverse events, but had an increased 30-day mortality.

### Reporting of weight-bearing status

Despite registries being firm proponents of early rehab and mobilization, many do not record WBS [13–19]. The Deutschen Gesellschaft für Unfallchirurgie (DGU) AltersTraumaRegister (ATR) [20] records weight-bearing status from the operation report, with 90% FWB in 2019. The Spanish Hip Fracture Registry also reports an unrestricted weight-bearing of 90% [21]. The ANZHFR cites FWB at 94% for New Zealand and 95% for Australia [7]. Our study demonstrated consistency with the national trend.

### Weight-bearing, fracture pattern, and operative intervention

Correlating RWB with fracture type and operation has not been published. The largest existing study by Ottesen et al. analyzed approximately 5000 patients with three coding entities consisting of (1) stabilization/hemiarthroplasty, (2) screw/side plate, and (3) cephalomedullary nail with external interpretation of these categories being difficult. Our study has shown that patients with RWB had a higher proportion of femoral neck fractures treated with impaction-type constructs (cannulated screws and sliding hip screws). Given the higher proportion of valgus-impacted fractures in the RWB category (38%), it could be argued that fixation should not be used if there is concern on the ability to load the construct. Impaction constructs have a reoperation rate of 20% over the course of 2 years [22], with 15 degrees of valgus angulation or posterior tilt significantly predictive of failure [23]. The two largest international hip fracture randomised control trials, FAITH and HEALTH, have demonstrated in sub-analyses that mortality, reoperation rate, and quality of life within 2 years favours arthroplasty over fixation in femoral neck fractures, despite patients receiving fixation being younger and healthier [24].

### Mortality

Ottesen et al. showed that 25% of the United States’ National Surgical Quality Improvement Program (NSQIP) patients were RWB, with 30-day mortality 5.5% for the RWB group and 3.2% in FWB. Using multivariate logistic regression, this remained a higher 30-day mortality risk for RWB (OR 1.77; 95% CI 1.31–2.41, *p* < 0.001). Whilst these 30-day mortality rates are substantially lower than our institution, approximately 10% of the Ottesen et al.’s patient sample were excluded due to medical issues, with this subset having a 21% 30-day mortality.

When mortality was examined for WBS using mediation, a total effect odds ratio of 0.42 (0.15–1.13) was calculated with only a 9% contribution from POD1mob. This, whilst non-significant, seems to indicate an association of RWB with decreased mortality risk which was not originally theorised, and dissimilar to the Ottesen et al. cohort [5]. This may simply be random chance or may be potentially due to selection bias towards patients who may be able to ‘cope’ with RWB. In the context of Government advocacy for FWB in all hip fractures, it is suspected that in our institution only those who can tolerate RWB, such as patients who are non-frail, previously independent in daily living, have family supports, and a comorbidity profile that ASA does not adequately account for, may be ordered RWB as they are considered ‘low-risk’ for immobility-related morbidity and mortality.

### Secondary outcomes

In a panel of immobility-related adverse events, RWB was related to DVTs which was not previously demonstrated [5]. The negative indirect effect infers other mediating factors not accounted for by the analysis. No other adverse events showed significance; however, the results need to be carefully interpreted due to small numbers and large confidence intervals.

RWB has been previously associated a higher risk of staying beyond 7 days (OR 1.69, 95% CI 1.47–1.95, *p* < 0.001), when 7 days is an arbitrary threshold, based on the 75th centile of LOS [5]. Our median institutional LOS was 7 days, and as a continuous variable, LOS did not show a difference with WBS. In the Australian setting, RWB has been studied in rehabilitation with an LOS of 34 (± 17) compared to 26 (± 11) in the FWB group (*p* = 0.04) [6].

RWB was not associated with an increase of revision surgery at 30 and 120 days. This does not seem to have been examined before in the literature. Return to the operating room within 30 days has been cited at 2.2% with no difference for RWB (OR 0.95, 95% CI 0.60–1.50, *p* = 0.814) [5]. Whilst this is not subcategorised into reason for return, it is similar to our 30-day reoperation rate (excluding infection) of 1.7% at 30-days. Wu et al. cited reoperation rates for failed fixation in their case series of 330 patients of 8 (3.1%) FWB and 5 (6.7%) RWB (*p* = 0.18); however, they neglected to include the length of follow-up, at what time these failures occurred, or the reason for fixation failure [6].

### Evidence for weight-bearing

The attitude of orthopaedic surgeons towards post-operative WBS has been previously explored. Despite awareness of ‘big data’ and standards, clinical acumen inevitably plays a role, and previous failure drives individual practice [25]. A potential deterrent for FWB is the relative paucity of data. Australian guidelines recommend immediate FWB, however, do not cite evidence [26], encouraging clinicians to: *“Allow patients to bear weight as tolerated, but avoid weight-bearing if there is a clinical concern about the fracture, the fixation or the likelihood of healing”*. Ambiguous statements such as this can be thus used to support restrictions.

The most comprehensive evidence-based guidelines regarding hip fracture are issued by the United Kingdom’s (UK) National Institute for Health and Care Excellence (NICE). In the 2017 document’s section examining full weight-bearing, it explicitly states: “There is no direct evidence relating to this recommendation in the immediate post-operative period, but the evidence from the early mobilization review question is indirectly applicable” [27].

The NSQIP Hip Fracture Targeted Procedure Dataset [5] demonstrated FWB was ordered for less than 75% of surgically treated patients. Whilst fracture patterns are not cited by the NSQIP, 23% of stabilization/hemiarthroplasty, 27% of screw/side plate, and 27% of cephalomedullary nails were not allowed FWB. Wu et al. published a series of hip fractures (*n* = 330) in rehabilitation, examined between 2003 and 2005, with a similarly high RWB proportion of 23% [6]. Despite the ANZHFR being instituted over a decade later, modern practice shows a different picture with participating hospitals having a contemporaneous mean RWB of 5%, despite outliers having over 20% RWB [7]. Wu demonstrated that patients had a longer LOS; however, no other outcomes were influenced by WBS, despite potentially being underpowered.

### Weight-bearing and post-operative day 1 mobilization

The opportunity to mobilize POD1 was realised in 81% of our cohort, similar to 59–89% of patients in other major registries [28]. Randomised evidence shows that hip fractures have functional and social benefit with early mobilization [29]. Early ambulation has additionally been shown to decrease pneumonia, delirium, length of stay [30], and inpatient mortality [31], whilst improving 6-month mortality [32] and reducing cost of hospitalisation [6]. The sub-analysis of patients not given the opportunity to mobilize POD1 demonstrated, they were generally less well and had increased 30-day mortality with adjustment. The small indirect mortality effect on WBS and that only 2.1% of patients who did not have the opportunity to mobilize POD1 were RWB implies that WBS restrictions are not a barrier for mobilizing.

In the 2017 NICE guidelines, the evidence linking mobilizing post-operatively to discharge destination (time and place) and mortality is described as low quality [27]. Unexpectedly, WBS and POD1mob were unrelated in this cohort. The specific patient factors determining why mobilization was not possible cannot be definitively identified retrospectively. The United Kingdom’s (UK) National Hip Fracture Database (NHFD) introduced the reason for patients not mobilizing POD1 as a new data point in 2020, which will help to develop strategies to mobilize post-operative patients [19]. In the NHFD, 79% of patients mobilized within 36 h of surgery [33]; these insights will most likely be applicable to many institutions.

Irrespective of WBS, not having the opportunity to mobilize POD1 was associated with higher mortality. This association with mortality has been well demonstrated [12, 34]. However, most of this evidence tends to be retrospective; hence, there is likely selection bias towards sicker patients being unable to mobilize or be mobilized by staff. Despite robust analyses and a detailed consideration of confounders, as this manuscript has attempted, biases in observational data may be difficult to adjust for, and the only randomised control trial looking at hip fractures and post-operative mobilization did not have mortality as an outcome[29].

### Limitations

Whilst WBS does not appear to affect mortality, mortality as an outcome may be considered a limitation of this study. Whilst death is irreversible and meaningful to clinicians, functional scores, and quality of life may be more meaningful to patients and families. A British study established that reduction of ‘the fear of falling for activity’ was the most valuable aspect of recovery for patients [35]; the DGU ATR records the ability to walk on POD7 [20]; the Swedish Rikshöft reports on the ability to mobilize outside at 120-days post fracture [18]; the Dutch Hip Fracture Audit looks at the KATZ-6 ADL score at 3 months [35, 36]; and the post-operative cumulative ambulatory score (CAS) is reported by the Irish Hip Fracture Database [15]. Whilst potentially more pragmatic outcomes, correlation of these outcomes with WBS is unknown.

The secondary outcome of acute orthopaedic length of stay has limited interpretation. As approximately 30% of patients were discharged to private rehabilitation, the LOS data do not accurately capture the full LOS within the health system. The binary nature of POD1mob is a weakness, and objective measure of how far a patient walked, or in what timeframe a set distance was ventured, may be of more clinical relevance.

We have mixed registry data with a comprehensive retrospective review to find clinically meaningful data that can highlight areas for change of practice, but there is inherent weakness and bias with retrospective reviews. The use of registry data is additionally problematic with considerable error rates in classification [37] despite the completeness of data for the ANZHFR being very high with moderate accuracy [38].

The heterogenous treatment modalities included in this cohort limit the application of the results. Whilst several operative interventions exist for each fracture type, it is recommended that all patients should be full weight-bearing, and this is the rationale for including all patients into the analysis. The ability for patients to comply with their prescribed weight-bearing status was also not assessed through objective means. Whilst patients who have restricted weight-bearing may be unable to comply with their limitations, conversely unrestricted weight-bearing may be compromised from post-operative pain or adverse events.

### Strengths

One of the strengths of this study is the prospective collection of patients that are truly geriatric hip fractures. The largest studies assessing both POD1 mobilization and WBS have been generated from the NSQIP [5, 12], which has been shown to have potential inaccuracies. In 2019, Shelton et al. from University of California Sacramento concluded “Caution should be taken when using NSQIP/TQIP databases to evaluate the care of GHFs” [39], on the background that 30% of patients presented as GHF by the NSQIP were not GHF when verified against an institutional database.

The ability to stratify the fractures that were most restricted by AO subclassification is also a strength. Nuances of fracture patterns influence fixation strategies, and subsequently, the willingness for surgeons to allow FWB. The AO classes of the common fracture patterns with RWB make sense, with future directions involving the education of surgeons to augment fixation or change surgical strategy (potentially to arthroplasty) if the fracture and construct do not allow FWB.

This study holds external validity as the patient demographics, fracture patterns, cognitive impairment, pre-fracture living, and mobility identified are very similar to the Australian [7] and international values [40]. Length of stay was similar to national average; however, 30-day mortality is close to the upper limits of predicted adjusted mortality for volume [7].

## Conclusion

In this study of post-operative geriatric hip fracture, restricted weight-bearing (RWB) was not associated with increased 30-day mortality in adjusted analysis. Not having the opportunity to mobilize post-operatively was predictive of higher 30-day mortality, however, only had a small effect on the relationship between RWB and mortality. From the immobility-related adverse events, only DVT was significantly higher among RWB patients. Neither length of stay nor reoperation at any time point was influenced by restricted weight-bearing status. We conclude that early mobilization may be more critical to mortality outcomes than unrestricted weight-bearing. Early mobilization is a target that is potentially easier to modify than surgical beliefs and technique. Whilst RWB may be necessitated in certain situations, patients should still be mobilized day 1 post-operatively, which appears to be associated with better outcomes. Further research should use larger datasets and causal modelling techniques to explore cause and effect rather than simple associations between these variables.

## Figures and Tables

**Figure:**
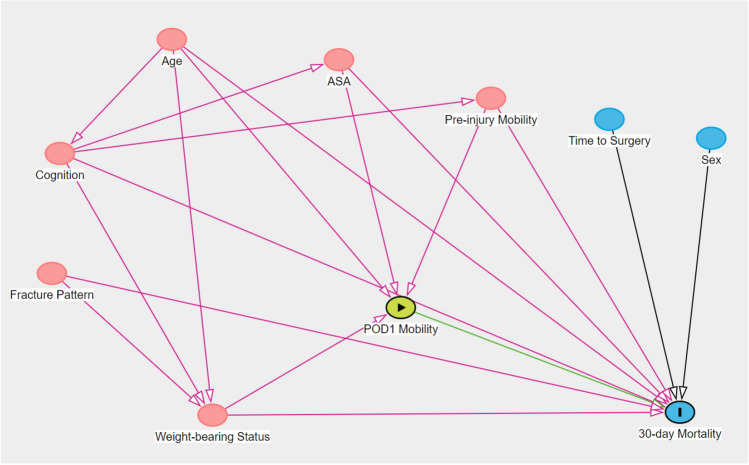
Appendix 1: Causal effect model of POD1mob (exposure) and 30-day mortality (outcome)

## Data Availability

Upon specific request pending appropriate approvals.

## Appendix

**Table:** Appendix 2: Comparison of day 1 mobilization demographics

Demographic	Opportunity to mobilize (*n* = 1160)	No opportunity to mobilize (*n* = 284)	*p* Value
Age (years; mean, SD)	83.4 (± 8.0)	85.0 (± 7.6)	< 0.001
Gender (female; *n*, %)	810 (69%)	192 (68%)	0.466
Residence (nursing home; *n*, %)	283 (24%)	85 (30%)	0.055
Mobility (*n*, %)			0.004
Independent (*n* = 630)	530 (46%)	100 (35%)	
One aid (*n* = 195)	159 (14%)	36 (13%)	
Frame (*n* = 576)	439 (38%)	137 (48%)	
Wheel chair/bed bound (*n* = 41)	30 (2.6%)	11 (3.9%)	
Cognitive impairment (impaired; *n*, %)	415 (36%)	145 (52%)	< 0.001
ASA score (score; *n*,%)			< 0.001
1	9 (0.8%)	2 (0.7%)	
2	195 (17%)	18 (6.5%)	
3	674 (59%)	161 (58%)	
4	257 (23%)	98 (35%)	
5	4 (0.3%)	0 (0%)	
Time to surgery (days; median, Q1,3)	1.02 (0.79–1.56)	1.02 (0.78–1.51)	0.843
Operations			0.125
Cannulated screws (*n* = 125)	111 (9.6%)	14 (4.9%)	
Cemented hemiarthoplasty (*n* = 419)	332 (29%)	87 (31%)	
Uncemented hemiarthroplasty (*n* = 27)	20 (1.7%)	7 (2.5%)	
Long femoral nail (*n* = 243)	186 (16%)	57 (20%)	
Short femoral nail (*n* = 491)	395 (34%)	96 (34%)	
Sliding hip screw (*n* = 44)	44 (3.8%)	9 (3.2%)	
Cemented total hip replacement (*n* = 75)	64 (5.5%)	11 (3.8%)	
Uncemented total hip replacement (*n* = 3)	3 (0.3%)	0 (0%)	
Weight-bearing status			0.365
Full weight-bearing	1111 (96%)	277 (98%)	
Restricted weight-bearing	49 (4.2%)	6 (2.1%)	

*ASA* American Society of Anesthesiologists, *SD* standard deviation

**Table:** Appendix 3: Multivariate analysis of day 1 mobilization

Outcome	Opportunity to mobilize (*n* = 1160)	No opportunity to mobilize (*n* = 284)	Exp(*b*)	95% CI	*p* value
Orthopaedic length of stay (days; median, Q1,Q3)^a^	7 (4–11)	7 (4–13)	1.10	(0.99–1.23)	0.084

			OR	95% CI	*p* value
Adverse events (type; *n*, %)^b^					
Pulmonary embolus	6 (0.5%)	3 (1.1%)	1.90	(0.43–8.37)	0.395
Deep vein thrombosis	7 (0.6%)	2 (0.7%)	1.29	(0.27–6.19)	0.753
Lower respiratory tract infection	95 (8.2%)	35 (12%)	1.27	(0.82–1.94)	0.283
Delirium	254 (22%)	81 (29%)	1.15	(0.83–1.58)	0.406
Urinary tract infection	194 (17%)	53 (19%)	1.03	(0.72–1.47)	0.864
Falls on the ward	13 (1.1%)	1 (0.35%)	0.25	(0.03–2.06)	0.200
30-Day mortality^b^	79 (6.8%)	51 (18%)	2.31	(1.53–3.48)	< 0.001

*ASA* American Society of Anesthesiology, *CI* confidence interval, *Exp(b)* exponential coefficient, *OR* odds ratio, *SE* standard error

^a^Generalised linear model using gamma regression adjusted for age, ASA score, pre-injury mobility, WBS, time to surgery

^b^Multivariate logistic regression adjusted for age, ASA score, pre-injury mobility, WBS, fracture type, time to surgery
